# Effects of procyanidin on cardiomyocyte apoptosis after myocardial ischemia reperfusion in rats

**DOI:** 10.1186/s12872-018-0772-x

**Published:** 2018-02-13

**Authors:** Dan Liu

**Affiliations:** Department of Cardiovascular Medicine, First Affiliated Hospital of Jinzhou Medical University, Renmin Street, Jinzhou, Liaoning Province 121000 China

**Keywords:** Ischemia reperfusion, Procyanidin, Signal transduction, Cardiomyocyte apoptosis

## Abstract

**Background:**

This study is aimed at investigating the effects of procyanidin on cardiomyocyte apoptosis of myocardial ischemia/reperfusion (I/R) injury in rats.

**Methods:**

Sprague-Dawley rats were randomly assigned into four groups: control group (normal saline), ischemic group (normal saline), procyanidin low-dose group (procyanidin 50 mg/kg/day) and procyanidin high-dose group (procyanidin 100 mg/kg/day) by intragastric administration for 2 weeks. After last administration, myocardial I/R model was induced by ligating left anterior descending artery for 30 min followed by 120 min of perfusion. The activity of serum creatine kinase mb isoenzyme (CK-MB) was detected after experiment. The content of reactive oxygen species (ROS) was determined by ROS fluorescent probe dihydroethidium; the expressions of p53, Caspase-9, Caspase-3, Bcl-2 and Bax were determined by western blotting; myocardial apoptosis was measured by the method of terminal deoxynucleotidyl transferase-mediated dUTP nick end labeling.

**Results:**

Compared with control group, the contents of serum CK-MB, ROS, the expressions of p53, Caspase-9, Caspase-3 and Bax increased significantly in ischemic group, the Bcl-2 expression, Bcl-2/Bax ratio decreased and the cardiomyocyte apoptosis index increased (*p* < 0.05); compared with ischemic group, the content of CK-MB, ROS, the expressions of p53, Caspase-9, Caspase-3 and Bax decreased, the Bcl-2 expression, Bcl-2/Bax ratio increased and the cardiomyocyte apoptosis index decreased in procyanidin group (*p* < 0.05).

**Conclusions:**

Procyanidin can reduce cardiomyocyte apoptosis after I/R. This beneficial effect is partially dependant on decreased ROS, p53, Caspase-9, Caspase-3 and Bax, as well as increased Bcl-2 and Bcl-2/Bax ratio.

## Background

Myocardial infarction is a major cause of death and disability worldwide [1]. Indeed, the use of thrombolytic therapy or primary percutaneous coronary intervention is the most effective strategy for reducing the size of a myocardial infarct and improving the clinical outcome. Unfortunately, restoring blood flow to the ischemic myocardium can also induce injury. It was coined the term of myocardial ischemia-reperfusion (I/R) injury [2]. How to protect against myocardial I/R injury has become a hot topic. The function of apoptosis in cardiovascular diseases is gaining recognition. Recent studies demonstrated that oxidative stress and I/R injury not only caused myocardial necrosis but also induced cardiomyocyte apoptosis [3]. Apoptosis is controlled by a series of physiological processes that mediate the signaling pathway; blocking the signaling pathway helps prevent myocardial apoptosis [3]. Procyanidins are the major group of polyphenols in the human diet because of their widespread occurrence in fruits, beans, cocoa-based products, wine, and beer [4, 5]. In recent years, the study of the physiological and pharmacological effects of procyanidins has been increasing. In the cardiovascular diseases, procyanidins provide several benefits, such as vasodilatation, antioxidation, improvement of endothelial function, anti-inflammatory and so on [6]. However, the effect of procyanidin on apoptosis has not been reported in myocardial infarction model. Therefore, the present study was designed to evaluate the effects of procyanidin on I/R and explore its potential mechanism.

## Methods

### Laboratory apparatus

DH-150-type animal respirator was purchased from Zhejiang Medical University Experimental Instrument Factory (Zhejiang, China). BIO-RAD electrophoresis tank was the product of BIO-RAD Company (California, USA). DNM-9602G ELISA analyzer, Shanghai Jinggong Engineering Co., Ltd. (Shanghai, China). 7170A automatic biochemical analyzer was purchased from Hitachi Company (Tokyo, Japan).

### Reagents

Procyanidin was obtained from Tianjin Jianfeng Natural Product R&D Co., Ltd. (purity > 95%; Tianjin, China). Terminal deoxynucleotidyl transferase-mediated dUTP nick end labeling (TUNEL) in situ apoptosis detection kit was purchased from Nanjing Key Gen Biotech. CO., LTD. (Nanjing, China). Rabbit polyclonal antibody against rat p-53, Caspase-9, Caspase-3, Bcl-2 and Bax was purchased from Biosynthesis Biotechnology Co., LTD. (Beijing, China); Reactive oxygen fluorescent probe dihydroethidium was obtained from Beyotime Institute of Biotechnology (Shenyang, China).

### Experimental animals

#### Ethical review

This study was carried out in strict accordance with the recommendations in the Guide for the Care and Use of Laboratory Animals of the National Institutes of Health. The protocol was approved by the Committee on the Ethics of Animal Experiments of the Jinzhou Medical University.

Healthy Sprague-Dawley (SD) rats (only male, 250-300 g) supplied by the experimental animal center of Jinzhou Medical University (certificate number: SCXK (Liao) 2003–007) were chosen for the current study.

### Methods

#### Experimental groups

Forty SD rats were randomly assigned to the following four groups (10 rats in each group): the control group (normal saline), ischemic group (normal saline), a low dose of procyanidin (50 mg/kg/day), and a high dose of procyanidin (100 mg/kg/day). The rats in all groups were continually intragastric administration for 2 weeks. All rats received the same volume. After 2 weeks, the rats except control group were made in vivo myocardial I/R models.

#### In vivo myocardial I/R model

We referenced the method of Ran et al. to do the myocardial I/R model [3, 7]. Briefly, male SD rats were anesthetized with 10% urethane (5 mg/kg). Catheter was inserted into the common carotid artery in order to take blood samples after the termination of experiment. After intubation, the animals were ventilated mechanically. The chest was opened, the pericardium incised, and the heart exposed. Under the atrial appendage, the beginning of the left anterior descending artery was circled with a 6.0 prolene suture. The ends of the suture were threaded through a piece of tubing, forming a snare that was tightened to occlude the artery. After the blood pressure and respiratory gradually stable (about 10-15 min), 30 min of coronary artery occlusion was operated. Tightening the snare occluded the coronary artery. Coronary artery occlusion was confirmed by epicardial cyanosis, ST-segment elevation, and T wave increasing. After 30 min of occlusion, the snare was released and the hearts were reperfused for 120 min. Reperfusion was achieved by releasing the snare and confirmed by recovery from cyanosis and sufficient ST-segment resolution (> 50%) [3, 8].

#### Serum examination

After the 120 min reperfusion period, to detect levels of serum creatine kinase mb isoenzyme (CK-MB), 1 mL of blood was collected through the carotid artery of live rats. Serum was separated immediately by centrifugation in 3000 rev min-1 for 10 min and stored at − 80 °C for use. The serum CK-MB was analyzed by the automatic biochemical analyzer. The assay was performed according to the manufacturer’s instructions.

#### Reactive oxygen species (ROS) determination in myocardium

One part of myocardium of left ventricle was fixed in 10% formalin and flash-frozen in liquid nitrogen and stored at − 80 °C for use. Then, this stored tissue was placed on the tissue support with optimum cutting temperature compound (OCT)-embedded. 10 μm tissue sections were cut and attached to the slides. Reactive oxygen fluorescent probe dihydroethidium was diluted to 10 umol/L by phosphate-buffered saline (PBS, pH 7.4), dropped to tissue and then incubated at 37 °C for 30 min, and then washed by PBS. Under the fluorescence microscope, N21 filter was selected to observe and shoot the image of cell red emission. ROS positive cells were stained red in the whole nucleus area. Mean absorbance was analyzed using Image-Pro Plus 6.0.

#### Western blot

One part of heart tissue (100 mg) were homogenized in liquid nitrogen and dissolved in 1 ml lysis buffer (Tris-HCl pH 7.5: 50 mmol/L; EDTA: 2 mmol/L; EGTA: 2 mmol/L; Aprotinin: 5 μg/mL; Pepstatin A: 5 μg/mL; Chymostatin: 5 μg/mL; Leupeptin: 5 μg/mL; DTT 1 mmol/L; Sodium deoxycholate: 5 mmol/L). The tissue was centrifuged in 15,000 rev min-1 at 4 °C for 30 min. We removed the pellet, and then took the supernatant to a fresh tube. BCA protein quantitative assay was used to determine protein concentrations. The protein lysates were loaded onto 10% SDS-polyacrylamide gel for separation, electrotransferred to Poly-vinylidene difluoride membranes, and blocked in 5% nonfat milk in tris-bufered saline. Membranes were incubated overnight using primary antibodies at 4 °C. After overnight incubation, membranes were washed for three times. This step was followed by secondary antibodies, which were conjugated using horseradish peroxidase and incubated for 1 h at room temperature. According to the manufacturer’s reagent instructions, the images were captured and documented. Intensities in the resulting bands were quantified by Image J image analysis system. Although identical amounts of protein were loaded into each well, the β-actin expression levels were used as a loading control and to normalize the results.

#### Apoptosis assay

One part of ischemic myocardium (left ventricle) was fixed in 10% formalin for 24 h and embedded with paraffin. Cardiomyocytes apoptosis were analyzed quantitatively by TUNEL staining with an in situ cell death detection kit. Based on the manufacturer’s instructions, all the procedures were performed. Cells were defined as apoptotic cells if the entire nuclear area of the cell was positively labeled. The apoptotic cells and bodies were counted in five high-power fields. The apoptotic index (AI) was calculated as the percentage of positively stained cells using the following equation: AI = number of apoptotic cells/total number of nucleated cells [9].

#### Statistical analysis

The results were expressed as means ± standard deviation (SD), analysis of variance (one-way ANOVA) was used to compare the control, ischemic and treatment groups. The SNK test was used to evaluate differences between two groups. All statistical analysis was performed using SPSS 16.0. A *P*-value < 0.05 was considered significant.

## Results

### Effects of myocardial I/R model on the serum levels of CK-MB

The serum levels of CK-MB were shown in Table 1. Compared with the control group, CK-MB in ischemic group increased (*P* < 0.05). Compared with the ischemic group, CK-MB in the low dose of procyanidin and the high dose of procyanidin groups decreased (*P* < 0.05).

**Table 1:** Effects of procyanidin on the serum level of CK-MB, the expression of ROS and cardiomyocyte apoptosis (*n* = 10 in each group)

group	CK-MB (IU/L)	ROS	AI (%)
control	978.94 ± 8.56	4.58 ± 0.67	4.31 ± 0.74
ischemic	5045.00 ± 7.83^★^	22.63 ± 1.60^★^	21.35 ± 3.10^★^
Procyanidin low-dose	3883.90 ± 10.70^▲^	16.84 ± 1.37^▲^	13.45 ± 2.14^▲^
Procyanidin high-dose	2907.30 ± 1.07^◆^	12.42 ± 0.95^◆^	9.43 ± 1.73^◆^

Note: Values are mean means ± standard deviation (SD). CK-MB: creatine kinase mb isoenzyme; ROS: reactive oxygen species; AI: apoptotic index. ^★^indicates compared with control group, *P*<0.05; ^▲^indicates compared with ischemic group, *P*<0.05; ^◆^indicates compared with procyanidin low-dose group, *P*<0.05

### Effects of procyanidin on ROS of myocardial I/R model

The effects of treatment for myocardial ischemia reperfusion model on ROS were shown in Table 1. Compared with the control group, ROS in ischemic group increased (*P* < 0.05). Compared with the ischemic group, ROS in the low dose of procyanidin and the high dose of procyanidin groups decreased (*P* < 0.01).

### Effects of procyanidin treatment for myocardial I/R model on p53, Caspase-9, Caspase-3, Bax and Bcl-2 expressions

**Fig. 1:**
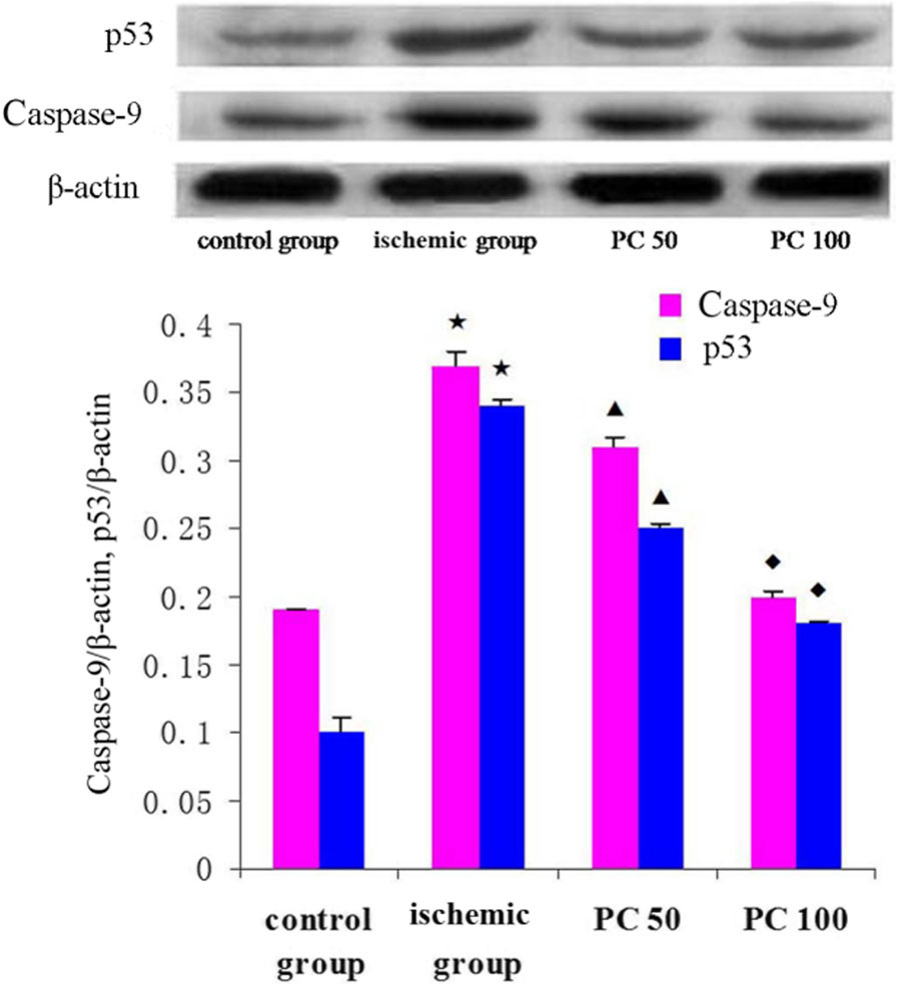
Effects of procyanidin on the expression of p53 and Caspase-9 protein level. PC 50: procyanidin 50 mg/kg/day; PC 100: procyanidin 100 mg/kg/day. ^★^ indicates compared with control group, *P*<0.05; ^▲^ indicates compared with ischemic group, *P*<0.05; ^◆^ indicates compared with procyanidin low-dose group, *P*<0.05

**Fig. 2:**
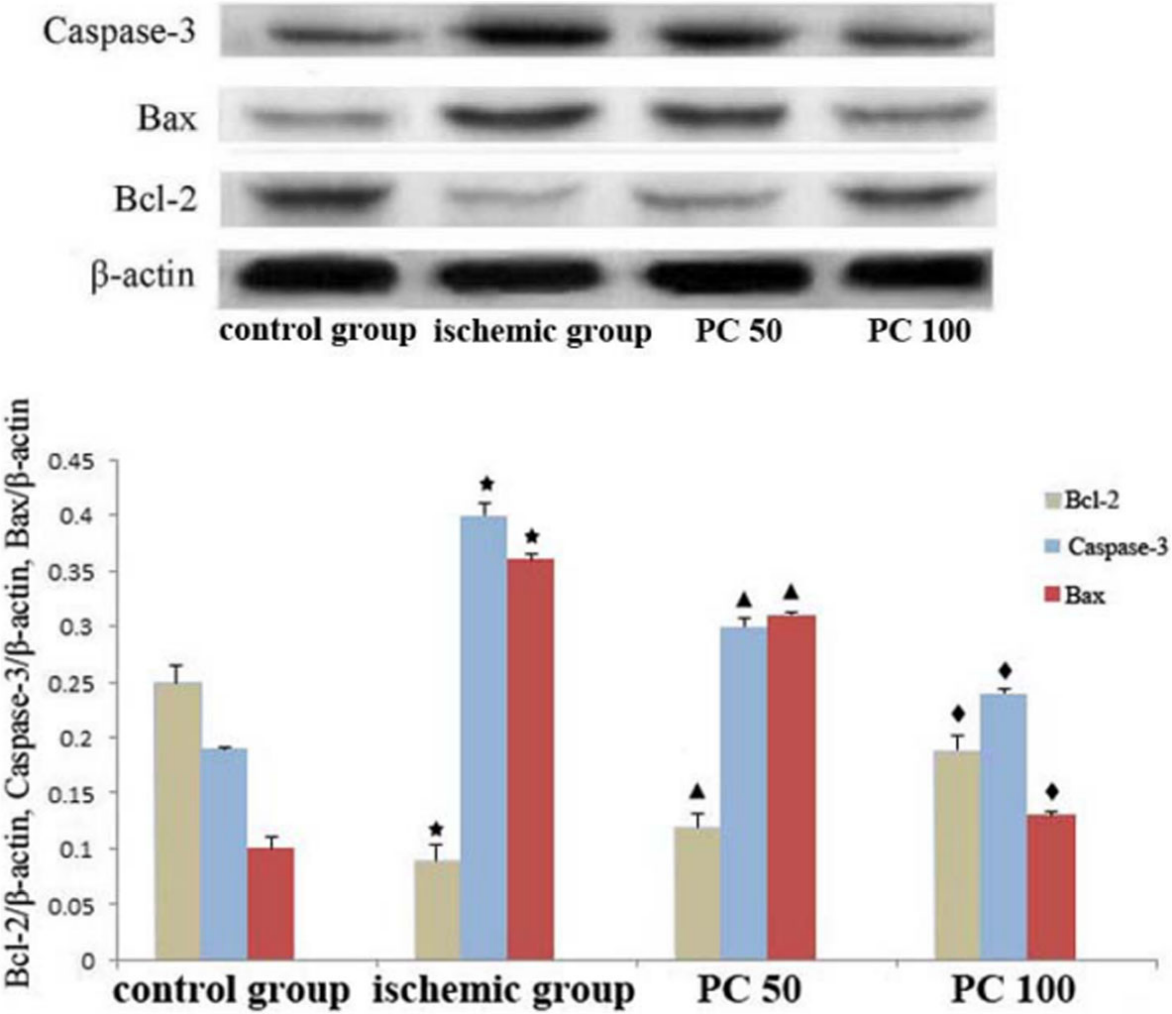
Effects of procyanidin on the expression of Caspase-3, Bax and Bcl-2 protein level. PC 50: procyanidin 50 mg/kg/day; PC 100: procyanidin 100 mg/kg/day. ^★^ indicates compared with control group, *P*<0.05; ^▲^ indicates compared with ischemic group, *P*<0.05; ^◆^ indicates compared with procyanidin low-dose group, *P*<0.05

**Fig. 3:**
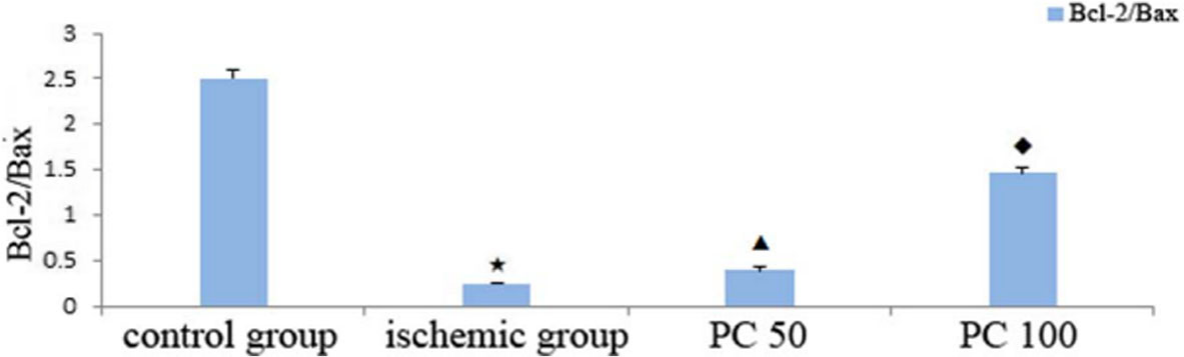
Effects of procyanidin on Bcl-2/Bax. PC 50: procyanidin 50 mg/kg/day; PC 100: procyanidin 100 mg/kg/day. ^★^ indicates compared with control group, *P*<0.05; ^▲^ indicates compared with ischemic group, *P*<0.05; ^◆^ indicates compared with procyanidin low-dose group, *P*<0.05

P53, Caspase-9, Caspase-3 and Bax expressions in the ischemic group increased, and Bcl-2 expression and Bcl-2/Bax ratio decreased than that in the control group (*p* < 0.05); Compared with the ischemic group, the low dose of procyanidin and the high dose of procyanidin groups decreased P53, Caspase-9, Caspase-3 and Bax expressions and increased Bcl-2 expression and Bcl-2/Bax ratio (*p* < 0.05) (Figs. 1, 2 and 3).

### Effects of procyanidin treatment for myocardial I/R model on myocardial apoptosis

**Fig. 4:**
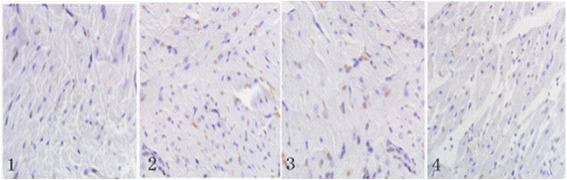
Effects of procyanidin on cardiomyocyte apoptosis in rats. 1. control group; 2. ischemic group; 3. procyanidin low-dose group; 4. procyanidin high-dose group

TUNEL staining suggested that more brown stained cells were found in the ischemic group than those in the control group (*p* < 0.05). Compared with the ischemic group, the low dose of procyanidin and the high dose of procyanidin groups decreased the number of apoptotic cells (*p* < 0.05). Effects of procyanidin treatment for myocardial I/R model on myocardial apoptosis were shown in Fig. 4 and Table 1.

## Discussion

The underlying pathophysiological mechanisms of I/R injury have not been fully elucidated [10]. It has been suggested that cell apoptosis, oxidative stress and calcium overload during the first minutes of reflow might be involved [10, 11]. Apoptosis is a process of normal cell death that maintains tissue homeostasis, but excessive apoptosis or its dysregulation can lead to various pathological processes, such as myocardial I/R injury [12]. Myocardial apoptosis accelerates the development of necrosis which might determine the degree of myocardial injury. How to reduce myocardial apoptosis is one of the hot spots in myocardial preservation. Oxidative stress is closely associated with cell apoptosis [13]. Reactive oxygen species ROS, including superoxide anion radicals, hydrogen peroxide, and hydroxyl radical, are produced from normal cellular metabolism process and some external factors such as exposure to agents known to cause oxidative stress. Physiological levels of ROS play an important role in intracellular signal transduction, follicle development, ovulation, and gene expression, while excessive ROS production leads to oxidative stress, which damages intracellular DNA, biomembrane lipids, proteins, and other macromolecules [14]. Accumulating evidence shows that the increased production of ROS has been related to vascular diseases, such as atherosclerosis, hypertension, diabetes vasculopathy, and restenosis. This increase seems to contribute to endothelial dysfunction, which is an early step of atherosclerosis [15]. At the beginning of myocardial I/R, a transient release of ROS is promptly generated which is responsible for a further reperfusion injury [16]. A great deal of research has showed that ROS generation may result in apoptosis during reperfusion injury, and reduced ROS generation can suppress reperfusion injury via activation of pathways [17].

Lysiak et al. [18] have investigated ROS in the mouse testis after I/R was mediated via a mitochondrial Caspase-9-dependent pathway involving the upstream mediators Caspase-2 and Bax. Shibata et al. [19] have demonstrated apoptosis in the neurodegenerative diseases was correlated with the activation of a ROS-p53-Caspase-9-mediated pathway. The ability of p53 to induce programmed cell death, or apoptosis, of cells exposed to environmental or oncogenic stress constituted a major pathway whereby p53 exerts its tumor suppressor function [20]. Liu et al. have found that removing ROS production significantly reduced p53, which exerting a positive feedback on ROS production [21]. P53 gene was an activator of cell apoptosis and inhibiting p53 gene significantly reduced cell apoptosis [22]. P53 can be transferred from the cytoplasm to the mitochondrial surface, ultimately leading to the endogenous mitochondrial dysfunction [23]. Endogenous mitochondrial dysfunction pathway was considered to be one of the major mechanisms responsible for I/R-induced cell death in ischemic heart [24].

Antiapoptotic members such as Bcl-2 family members play essential roles in apoptosis caused by all kinds of stimulus signals. Bcl-2 and Bax play important pathophysiological roles in the protection and acceleration, respectively, of myocyte apoptosis after ischemia and/or reperfusion [25]. Bcl-2 prevents death that occurs through the intrinsic apoptotic pathway by preventing the loss of outer mitochondrial membrane integrity. Bax, a member of the bcl-family, homodimerizes and forms heterodimers with bcl-2 protein, reducing its anti-apoptotic effect. The balance of Bcl-2 and Bax in apoptotic cells is directly related to the regulation of apoptosis: the increase in Bax levels promotes cell apoptosis, whereas Bcl-2 increases the inhibition of cell apoptosis. The Bcl-2/Bax ratio determines the viability of cells after apoptotic stimulation [26]. When the Bcl-2/Bax ratio increases, the apoptosis rate is inhibited. And the decreased Bcl-2/Bax ratio leads to apoptosis. Caspases-activated mitochondrial pathway is a definitely regulatory mechanism of apoptosis. In the Caspases-activated mitochondrial pathway, the Caspases family is responsible for important apoptosis regulation. Caspases-3 is the most important apoptotic performer. Bcl-2 blocks the activation of upstream Caspases by interfering with the cytochrome c release and inhibits cell apoptosis [27]. Increases in p53 and Bax expressions may lead to mitochondrial depolarization and cytochrome c release [25]. Cytochrome c and apoptosis protease activation factor 1 (Apaf-1) and procaspase-9 binded and formed apoptotic bodies and then further activated procaspase-9 into Caspase-9, resulting in the downstream activation of executioner Caspases (Caspase-3, 6, 7) to augment apoptosis [28, 29]. Our results indicate, compared with the ischemic group, the low dose of procyanidin and the high dose of procyanidin groups decreased p53, Caspase-9, Caspase-3 and Bax expressions and increased Bcl-2 protein expression as well as Bcl-2/Bax ratio, especially in high dose of procyanidin group. Although we have identified some mechanisms of procyanidin-mediated cardioprotection, these are not necessarily the only, or even the most important, effects. It still requires a great number of animal experiments in order to verify the mechanism of procyanidin-mediated cardioprotection.

Our study also demonstrates that procyanidin effectively protects cardiac myocytes against I/R injury. Compared with the ischemic group, the low dose of procyanidin and the high dose of procyanidin groups decreased the number of apoptotic cells, serum CK-MB level. Thereby we proposed that procyanidin protected against reperfusion injury-induced cell apoptosis with a dose-dependent.

## Conclusion

In summary, our study shows that procyanidin ameliorated reperfusion injury by exerting an anti-apoptotic effect on rats. This beneficial effect is partially dependant on decreased ROS, p53, Caspase-9, Caspase-3 and Bax expressions, increased Bcl-2 expression and Bcl-2/Bax ratio. The present study provides experimental evidence to merit further exploration of the possible use of procyanidin, as a therapeutic approach in the treatment of I/R injury on rats. It has only begun with a prologue for a new research and still requires a great number of experiments in order to verify the novel therapeutic approaches to ischemic injury.

## Abbreviations

CK-MB: Creatine kinase mb isoenzyme
I/R: Ischemia/reperfusion
OCT: Optimum cutting temperature compound
PBS: Phosphate-buffered saline
ROS: Reactive oxygen species
SD: Sprague-Dawley
SD: Standard deviation
TUNEL: Terminal deoxynucleotidyl transferase-mediated dUTP nick end labeling

## References

[1] Thygesen K, Alpert JS, Jaffe AS, Simoons ML, Chaitman BR, White HD, Thygesen K, Alpert JS, White HD, Jaffe AS, Katus HA, Apple FS, Lindahl B, Morrow DA, Chaitman BA, Clemmensen PM, Johanson P, Hod H, Underwood R, Bax JJ, Bonow RO, Pinto F, Gibbons RJ, Fox KA, Atar D, Newby LK, Galvani M, Hamm CW, Uretsky BF, Steg PG, Wijns W, Bassand JP, Menasché P, Ravkilde J, Ohman EM, Antman EM, Wallentin LC, Armstrong PW, Simoons ML, Januzzi JL, Nieminen MS, Gheorghiade M, Filippatos G, Luepker RV, Fortmann SP, Rosamond WD, Levy D, Wood D, Smith SC, Hu D, Lopez-Sendon JL, Robertson RM, Weaver D, Tendera M, Bove AA, Parkhomenko AN, Vasilieva EJ, Mendis S, Writing Group on the Joint ESC/ACCF/AHA/WHF Task Force for the Universal Definition of Myocardial Infarction, ESC Committee for Practice Guidelines (CPG) (2012). Third universal definition of myocardial infarction. Eur Heart J.

[2] Jones DA, Andiapen M, Van-Eijl TJ, Webb AJ, Antoniou S, Schilling RJ, Ahluwalia A, Mathur A (2013). The safety and efficacy of intracoronary nitrite infusion during acute myocardial infarction (NITRITE-AMI): study protocol of a randomized controlled trial. BMJ Open.

[3] Ran X, Diao JX, Sun XG, Wang M, An H, Huang GQ, Zhao XS, Ma WX, Zhou FH, Yang YG, Miao CM (2015). Huangzhi oral liquid prevents arrhythmias by Upregulating Caspase-3 and apoptosis network proteins in myocardial ischemia-reperfusion injury in rats. Evid Based Complement Alternat Med.

[4] Cedó L, Castell-Auví A, Pallarès V, Macià A, Blay M, Ardévol A, Motilva MJ, Pinent M (2014). Gallic acid is an active component for the anticarcinogenic action of grape seed procyanidins inpancreatic cancer cells. Nutr Cancer.

[5] Bai H, Wang Z, Cui J, Yun K, Zhang H, Liu RH, Fan Z, Cheng C (2014). Synergistic radiation protective effect of purified Auricularia auricular-judae polysaccharide (AAP IV) with grape seed procyanidins. Molecules.

[6] Wang CC, Huang PL, Liu TY, Jan TR (2009). Highly oligomeric procyanidins from areca nut induce lymphocyte apoptosis via the depletion ofintracellular thiols. Toxicol in Vitro.

[7] Hale SL, Kloner RA (2010). Cardioprotection with adenosine-regulating agent, GP531: effects on no-reflow, infarct size, and blood flow following ischemia/reperfusion in the rabbit. J Cardiovasc Pharmacol Ther.

[8] Steg PG, James SK, Atar D, Badano LP, Blömstrom-Lundqvist C, Borger MA, Di Mario C, Dickstein K, Ducrocq G, Fernandez-Aviles F, Gershlick AH, Giannuzzi P, Halvorsen S, Huber K, Juni P, Kastrati A, Knuuti J, Lenzen MJ, Mahaffey KW, Valgimigli M, van’t Hof A, Widimsky P, Zahger D (2012). ESC guidelines for the management of acute myocardial infarction in patients presenting with ST-segment elevation. Eur Heart J.

[9] Jyothi P, Riyaz N, Nandakumar G, Binitha MP (2008). A study of oxidative stress in paucibacillary and multibacillary leprosy. Indian J Dermatol Venereol Leprol.

[10] Moens AL, Claeys MJ, Timmermans JP, Vrints CJ (2005). Myocardial ischemia/reperfusion-injury, a clinical view on a complex pathophysiological process. Int J Cardiol.

[11] Sun L, Fan H, Yang L, Shi L, Liu Y (2015). Tyrosol prevents ischemia/reperfusion-induced cardiac injury in H9c2 cells: involvement of ROS, Hsp70, JNK and ERK, and apoptosis. Molecules.

[12] Kim YJ, Kim YA, Yokozawa T (2011). Pycnogenol modulates apoptosis by suppressing oxidative stress and inflammation in high glucose-treated renal tubular cells. Food Chem Toxicol.

[13] Yu D, Li M, Tian Y, Liu J, Shang J (2015). Luteolin inhibits ROS-activated MAPK pathway in myocardial ischemia/reperfusion injury. Life Sci.

[14] Zhang JQ, Gao BW, Wang J, Ren QL, Chen JF, Ma Q, Zhang ZJ, Xing BS (2016). Critical role of FoxO1 in Granulosa cell apoptosis caused by oxidative stress and protective effects of grape seed Procyanidin B2. Oxidative Med Cell Longev.

[15] Álvarez E, Rodiño-Janeiro BK, Jerez M, Ucieda-Somoza R, Núñez MJ, González-Juanatey JR (2012). Procyanidins from grape pomace are suitable inhibitors of human endothelial NADPH oxidase. J Cell Biochem.

[16] Van Wijk SJ, Hageman GJ (2005). Poly (ADP-ribose) polymerase-1 mediated caspase-independent cell death after ischemia/reperfusion. Free Radic Biol Med.

[17] Wang X, Cui L, Joseph J, Jiang B, Pimental D, Handy DE, Liao R, Loscalzo J (2012). Homocysteine induces cardiomyocyte dysfunction and apoptosis through p38 MAPK-mediated increase in oxidant stress. J Mol Cell Cardiol.

[18] Lysiak JJ, Zheng S, Woodson R, Turner TT (2007). Caspase-9-dependent pathway to murine germ cell apoptosis: mediation by oxidative stress, BAX, and caspase 2. Cell Tissue Res.

[19] Shibata N, Kobayashi M (2008). The role for oxidative stress in neurodegenerative diseases. Brain Nerve.

[20] Pietsch EC, Sykes SM, McMahon SB, Murphy ME (2008). The p53 family and programmed cell death. Oncogene.

[21] Liu H, Pedram A, Kim JK (2011). Oestrogen prevents cardiomyocyte apoptosis by suppressing p38α-mediated activation of p53 and by down-regulating p53 inhibition on p38β. Cardiovasc Res.

[22] Liu P, Xu B, Cavalieri TA, Hock CE (2006). Pifithrin-alpha attenuates p53-mediated apoptosis and improves cardiac function in response to myocardial ischemia/reperfusion in aged rats. Shock.

[23] Endo H, Saito A, Chan PH (2006). Mitochondrial translocation of p53 underlies the selective death of hippocampal CA1 neurons after global cerebral ischaemia. Biochem Soc Trans.

[24] Alam MR, Baetz D, Ovize M (2015). Cyclophilin D and myocardial ischemia-reperfusion injury: a fresh perspective. J Mol Cell Cardiol.

[25] Babu PP, Suzuki G, Ono Y, Yoshida Y (2004). Attenuation of ischemia and/or reperfusion injury during myocardial infarction using mild hypothermia in rats: an immunohistochemical study of Bcl-2, Bax, Bak and TUNEL. Pathol Int.

[26] McClintock DS, Santore MT, Lee VY, Brunelle J, Budinger GR, Zong WX, Thompson CB, Hay N, Chandel NS (2002). Bcl-2 family members and functional electron transport chain regulate oxygen deprivation-induced cell death. Mol Cell Biol.

[27] Xie Z, Koyama T, Suzuki J, Fujii Y, Togashi H, Sawa H, Nagashima K (2001). Coronary reperfusion following ischemia: different expression of bcl-2 and bax proteins, and cardiomyocyte apoptosis. Jpn Heart J.

[28] Kadenbach B, Arnold S, Lee I, Hüttemann M (2004). The possible role of cytochrome c oxidase in stress-induced apoptosis and degenerative diseases. Biochim Biophys Acta.

[29] Zou H, Li Y, Liu X, Wang X (1999). An APAF-1. Cytochrome c multimeric complex is a functional apoptosome that activates procaspase-9. J Biol Chem.

